# A Bayesian network analysis of posttraumatic stress disorder symptoms in adults reporting childhood sexual abuse

**DOI:** 10.1080/20008198.2017.1341276

**Published:** 2017-07-15

**Authors:** Richard J. McNally, Alexandre Heeren, Donald J. Robinaugh

**Affiliations:** ^a^ Department of Psychology, Harvard University, Cambridge, MA, USA; ^b^ Institute of Psychological Science, Université Catholique de Louvain, Louvain-la-Neuve, Belgium; ^c^ Department of Psychiatry, Massachusetts General Hospital and Harvard Medical School, Boston, MA, USA

**Keywords:** Network analysis, directed acyclic graph, PTSD, childhood sexual abuse

## Abstract

**Background:** The network approach to mental disorders offers a novel framework for conceptualizing posttraumatic stress disorder (PTSD) as a causal system of interacting symptoms.

**Objective:** In this study, we extended this work by estimating the structure of relations among PTSD symptoms in adults reporting personal histories of childhood sexual abuse (CSA; N = 179).

**Method:** We employed two complementary methods. First, using the graphical LASSO, we computed a sparse, regularized partial correlation network revealing associations (edges) between pairs of PTSD symptoms (nodes). Next, using a Bayesian approach, we computed a directed acyclic graph (DAG) to estimate a directed, potentially causal model of the relations among symptoms.

**Results:** For the first network, we found that physiological reactivity to reminders of trauma, dreams about the trauma, and lost of interest in previously enjoyed activities were highly central nodes. However, stability analyses suggest that these findings were unstable across subsets of our sample. The DAG suggests that becoming physiologically reactive and upset in response to reminders of the trauma may be key drivers of other symptoms in adult survivors of CSA.

**Conclusions:** Our study illustrates the strengths and limitations of these network analytic approaches to PTSD.

The ontology of posttraumatic stress disorder (PTSD) has been a subject of considerable controversy (McNally, 2012). One controversy concerns whether PTSD is a socially constructed idiom of distress (e.g. Summerfield, 2001) or a natural kind akin to diseases that appear throughout the world and that have afflicted humanity throughout history (e.g. Osterman & de Jong, 2007). Another concerns whether PTSD is best characterized as a discrete, categorical entity (American Psychiatric Association [APA], 2013), or as a continuum of stress responsiveness (Ruscio, Ruscio, & Keane, 2002).

Until recently, ontological discourse on PTSD was largely confined to social constructionist versus natural kind, and categorical (discrete) versus dimensional (continuum) debates (McNally, 2017). The network perspective on psychopathology, pioneered by Borsboom and his associates (e.g. Borsboom, 2008, 2017; Borsboom & Cramer, 2013; Cramer, Borsboom, Aggen, & Kendler, 2012; Cramer, Waldorp, van der Maas, & Borsboom, 2010), offers a radically different way of understanding PTSD (McNally, 2012) and other mental disorders (for reviews, see Fried et al., 2017; McNally, 2016). According to this perspective, a mental disorder is not an underlying, latent (unobserved) disease entity, whether construed categorically or dimensionally. Rather, it is an emergent phenomenon, arising from causal interactions among its constitutive symptoms. That is, the symptoms of PTSD do not cohere syndromically because they share an underlying cause, but rather because of causal interactions among the symptoms themselves. The relation of symptoms to disorder is not one of cause and effect; it is *mereological* – part(s) to whole (Borsboom, 2008).

The network framework resembles Boyd’s (1991) *homeostatic property cluster kinds*, a nonessentialist conceptualization of biological species justly celebrated by clinicians as relevant to psychopathology (Kendler, Zachar, & Craver, 2011; McNally, 2011, p. 206). According to Boyd, a species signifies a natural kind comprising a cluster of properties that hang together despite environmental perturbations, not an underlying essence shared by each member of a species. According to Borsboom, a disorder signifies a cluster of symptoms that hang together in virtue of their functional interrelations, not an underlying, latent essence that causes symptom emergence and covariation. However, the network approach transcends conceptual models, such as Boyd’s, by furnishing powerful computational methods for modelling and visualizing disorders as symptom networks.

The network approach not only offers a novel framework for conceptualizing and tools for studying PTSD; it also illuminates new avenues of research that will advance our understanding of the disorder. Most notably, it calls for further research exploring the relations among PTSD symptoms. This focus is a marked departure from traditional categorical and dimensional latent construct approaches. Indeed, to justify an inference to a latent common cause, these approaches must presuppose that no causal interactions occur among the symptoms themselves – a highly implausible supposition (Borsboom, 2008). As every clinician knows, symptoms of mental disorders interact with one another (e.g. obsessions motivate compulsions; insomnia causes fatigue), thereby violating the axiom of local independence requisite to this inference. Rather than forbidding such interactions, the network approach embraces these causal relations among symptoms. Indeed, much of the early work applying network analysis to stress-related syndromes characterized them as potentially causal systems of interacting symptoms (e.g. PTSD in earthquake survivors, McNally et al., 2015; complicated grief following spousal bereavement, Robinaugh, LeBlanc, Vuletich, & McNally, 2014; Robinaugh, Millner, & McNally, 2016).

In the study reported here, we extended this work by estimating the structure of relations among PTSD symptoms in adults reporting personal histories of childhood sexual abuse (CSA). To accomplish this objective, we used two distinct approaches to network analysis to characterize the associations (edges) between symptoms (nodes). First, we used the graphical LASSO (Least Absolute Shrinkage and Selection Operator; Friedman, Hastie, & Tibshirani, 2008) algorithm to produce a regularized partial correlation network whose edges represent the magnitude of association between pairs of symptoms after adjusting for the influence of all other symptoms. The purpose in doing so is to identify potential causal relationships that may figure into the disorder’s aetiology and maintenance. However, the resultant network is undirected and therefore cannot tell us whether an edge connecting symptom X and symptom Y means that activation of symptom X predicts the activation of symptom Y (or vice versa) or whether the direction of prediction (and possibly causation) occurs both ways.

Accordingly, to further investigate the causal structure of the PTSD network, we used a Bayesian approach to compute a directed acyclic graph (DAG) that best captures the conditional independence relations among PTSD symptoms. This analysis not only detects direct associations between pairs of symptoms, but also estimates the direction of the association and hence suggests hypotheses about potentially causal relations (Pearl, Glymour, & Jewell, 2016).

In summary, we used two distinct approaches to model PTSD symptoms as networks comprising direct, and potentially causal, relations between symptom pairs. First, we computed an undirected, regularized partial correlation graph – the most popular method for modelling psychopathology (Epskamp & Fried, 2016). Next, we used Bayesian methods to compute a directed, acyclic graph – a relatively new approach to modelling psychopathology (McNally, Mair, Mugno, & Riemann, 2017). Both approaches test for links between symptoms after adjusting for the influence of other symptoms, but their strengths and weaknesses are mirror images of one another. The first network comprises undirected edges; hence one cannot tell whether symptom X predicts (or possibly causes) symptom Y, vice versa, or both. However, it has the advantage of illuminating potential causal loops among symptoms. The second network is directed such that the arrow of prediction is evident, potentially illuminating the direction of causality. However, that influence is restricted to travelling in only one direction. That is, activation originating in a node cannot return to its node of origin, as in a positive feedback cycle. This is a limitation because at least some psychopathology networks are likely to contain the very cycles forbidden by DAGs.

By using data from adults reporting histories of CSA, we applied network analysis to a new traumatized group beyond the earthquake survivors (McNally et al., 2015), war veterans (Armour, Fried, Deserno, Tsai, & Pietrzak, 2017; Mitchell et al., 2017), and civilian accident and assault survivors (Bryant et al., 2017) that have been the focus of prior studies. Hence, in contrast to previous PTSD studies, our current study involved participants whose stressors were always interpersonal, sexual, and occurred many years earlier.

## 1. Method

### 1.1. Participants

The de-identified archival data came from adults who reported having been sexually abused during childhood and who had enrolled in two research programmes approved by Harvard University’s Committee on the Use of Human Subjects. The first programme concerned cognitive functioning (e.g. McNally, Clancy, Barrett, & Parker, 2005; McNally, Ristuccia, & Perlman, 2005), and the second programme concerned risk and resilience in survivors of CSA (e.g. McNally & Robinaugh, 2011; Robinaugh & McNally, 2011). Participants were from the Boston area, recruited via newspaper advertisements. To qualify as a CSA survivor, potential participants had to have had experienced at least one episode of CSA prior to the age 16 that involved unwanted physical contact (e.g. fondling; oral, anal, or vaginal penetration) involving a perpetrator at least five years older than the victim.

The participants from the first research programme comprised 89 participants (women = 60, men = 29) whose mean age was 40.1 years (*SD* = 12.5). Inputs for the network analyses were 17 PTSD symptoms (APA, 1994) from the PTSD Symptom Scale-Interview Version (PSS; Foa & Tolin, 2000) whereby the assessor probes for the frequency and severity of each symptom in reference to the previous two weeks, and rates it on a 4-point scale ranging from 0 (‘not at all’) through 3 (‘5 or more times per week/very much’). The interviewer informed participants that CSA was the referent event for answering questions about PTSD.

The participants from the second research programme comprised 90 women whose mean age was 42.2 years (*SD* = 12.2). Accordingly, the mean age of the 179 CSA participants was 41.2 years (*SD* = 12.4). For these participants, symptom ratings came from the Posttraumatic Checklist-Civilian (PCL-C) version (Weathers, Litz, Herman, Huska, & Keane, 1993). For this self-report questionnaire, the participant rates each PTSD symptom on a 5-point scale ranging from one (*Not at all*) to five (*Extremely*) to indicate how much they have been bothered by it during the past month. The research team informed participants that CSA was the referent event for answering questions on the PCL-C.

Although the interview and the questionnaire both assessed the same 17 PTSD symptoms, the time frames were slightly different as were the scales. Therefore, we rescaled the PCL-C by converting 1 to 0, 2 to 1, 3 to 2, and converting 4 and 5 to 3. Hence, we collapsed the two most extreme points on the PCL-C to the single most extreme point on the PSS.

### 1.2. Network analyses

#### 1.2.1. Regularized partial correlation network

We used a Graphical Gaussian Model (GGM) to estimate a network consisting of regularized partial correlations between symptom pairs (Epskamp & Fried, 2016). A GGM is a type of pairwise Markov random field (MRF) suitable for modelling metric data. A MRF comprises a set of random variables characterized by the Markov property and depictable as an undirected network. A stochastic process is Markovian if the conditional probability distribution of the states of the process depends only on its current state, not on events that occurred prior to its current state. The ordinality of the PSS and PCL-C notwithstanding, we assume that the clinical phenomena they tap are metric (e.g. *frequency* of flashbacks), hence justifying a GGM.

We regularized our model by running the graphical LASSO (Friedman et al., 2008) algorithm via the R packages *glasso* (Friedman, Hastie, & Tibshirani, 2014) and *qgraph* (Epskamp, Cramer, Waldorp, Schmittmann, & Borsboom, 2012). The aim of this procedure is to compute a parsimonious (‘sparse’) network that accounts for the most variance with the fewest number of edges. Because our PTSD symptoms are ordinal variables, we calculated the polychoric correlations among symptoms before conducting the regularization.

The *glasso* package drives small partial correlations to zero such that edges of such trivial magnitude disappear from the network. The *qgraph* package provides an Extended Bayesian Information Criterion (EBIC) that ascertains the tuning parameter lambda (λ) that optimizes model fit as well as parsimony (Chen & Chen, 2008), given an investigator-provided value of the hyperparameter gamma (γ). Lambda values can range from zero, whereby every edge remains in the network, to a value equivalent to the largest absolute correlation, whereby no edge remains in the network. Lower values of λ result in heightened sensitivity to detect genuine edges, but at the risk of retaining spurious ones (‘false alarms’), whereas higher values of λ result in heightened specificity, but at the risk of excluding genuine edges. The resultant sparse graph depicts the largest and likely genuine direct symptom–symptom connections that cannot be attributed to the influence of other symptoms in the network.

The hyperparameter gamma (γ) is usually set between zero and 0.5 (Epskamp & Fried, 2016). The closer γ is to 0.5, the more the EBIC will favour a simpler model containing fewer edges, increasing confidence that the edges are genuine (i.e. high specificity). The closer γ is to zero, the more the EBIC will favour a model with more edges (i.e. high sensitivity). Following Beard et al. (2016), we set γ to 0.5.

To quantify the importance of each node in the network, we computed centrality indices (Freeman, 1978/1979; Opsahl, Agneessens, & Skvoretz, 2010). Centrality indices reflect how connected a node is within the network and hence how potentially clinically relevant it may be. The *betweenness* centrality of a node equals the number of times that it lies on the shortest path between any pair of other nodes. *Closeness* centrality signifies the average distance of a node to all other nodes in the network, calculated as the inverse of the weighted sum of shortest path lengths of a given node to reach all other nodes network. Node *strength* is the sum of the absolute value of the edge weights connected to a node.

Of the three centrality indices, node strength may be the most relevant index of importance for modelling symptom networks (as opposed to, for example, airline networks where nodes signify airports, edges signify flight routes, and centrality indices such as betweenness signify the importance of an individual airport as a hub within the network). An episode of disorder is more likely to occur if a stressor activates a symptom having high strength centrality than if it activates a symptom having low strength centrality as the former has more and stronger links to other symptoms than does the latter. Using the R package *qgraph* (Epskamp et al., 2012), we computed all three centrality metrics, and plotted the normalized (*z*-scored) values for each node.

We evaluated the robustness of our findings by using the *R* package *bootnet* (Epskamp, Borsboom, & Fried, 2016). We first estimated the accuracy of the edge weights by employing a non-parametric bootstrap approach to calculate the 95% confidence intervals (CIs) for the edges by sampling the data 10,000 times (with replacement), thereby generating a distribution of edge weights.

We then evaluated the stability of the centrality metrics by using a subset bootstrap procedure (Costenbader & Valente, 2003), whereby we repeatedly correlated centrality metrics of the original data set with those calculated from subsamples comprising progressively fewer participants. To quantify the effects of this person-dropping procedure, we calculated the centrality stability correlation coefficient (CS-coefficient). The CS-coefficient represents the maximum proportion of participants that can be dropped while maintaining 95% probability that the correlation between centrality metrics from the full data set and the subset data are at least .70. CS-coefficients above .50 indicate stable centrality metrics and a minimum CS-coefficient of .25 is recommended for interpreting centrality indices.

Finally, we computed the correlation between the standard deviation and the strength centrality for the 17 PTSD symptoms to test whether differential variability (‘restricted range’) in symptom severity ratings may have distorted conclusions about symptom importance (see Fried & Nesse, 2014; Terluin, De Boer, & De Vet, 2016). We also computed the skewness for each symptom as well as the correlation between skewness and strength centrality.

#### 1.2.2. Directed acyclic graph (DAG)

To compute a Bayesian network, visualized as a DAG, we ran the *hill-climbing* algorithm furnished by the R package *bnlearn* (Scutari, 2010). The bootstrap function of *bnlearn* calculates the structural aspect of the network by adding edges, subtracting them, and reversing their direction to optimize the Bayesian Information Criterion (BIC) – a goodness-of-fit target score. This first step ascertains whether an edge between two symptoms exists; it does not determine its weight. We then randomly restarted this procedure with various candidate edges potentially linking different pairs of symptoms, perturbed the system, and so forth. As this iterative process unfolds, the algorithm discerns the network’s structure. Because we had a missing data point for at least one of the 17 symptoms for 14 participants, our Bayesian analysis involved 165 rather than 179 participants.

To ensure the stability of the DAG, we bootstrapped 10,000 samples, computing a network for each sample (McNally et al., 2017). We then averaged them to obtain the final, resultant network. There are two steps involved. First, we determined how often an edge appeared in the 10,000 bootstrapped networks. We then used Scutari and Nagarajan’s (2013) statistically-driven method for retaining edges in the final, averaged network. Their method yields networks having high sensitivity as well as high specificity. Second, we determined the direction of each edge in each of the 10,000 bootstrapped networks. If an edge pointed from symptom X to symptom Y in at least 51% of the networks, then this direction was depicted in the final, averaged network.

We visualized the final, averaged network in two ways. First, we computed a DAG whose edges depicted the BIC value of an edge. High absolute BIC values signify the importance of an edge to the model that best captures the structure of the data. Edge thickness depicts the magnitude of the BIC value. The thicker an edge, the more damaging it would be to model fit if the edge were removed from the network. Second, we computed a DAG whereby edge thickness signifies the probability that the edge points in the direction depicted. Hence, if an edge pointed from symptom X to symptom Y in 9,950 of 10,000 bootstrapped networks, it would appear very thick. If it pointed from symptom X to symptom Y in only 5,500 of 10,000 bootstrapped networks, it would appear very thin.

## 2. Results

### 2.1. Regularized partial correlation network

**Figure 1.**
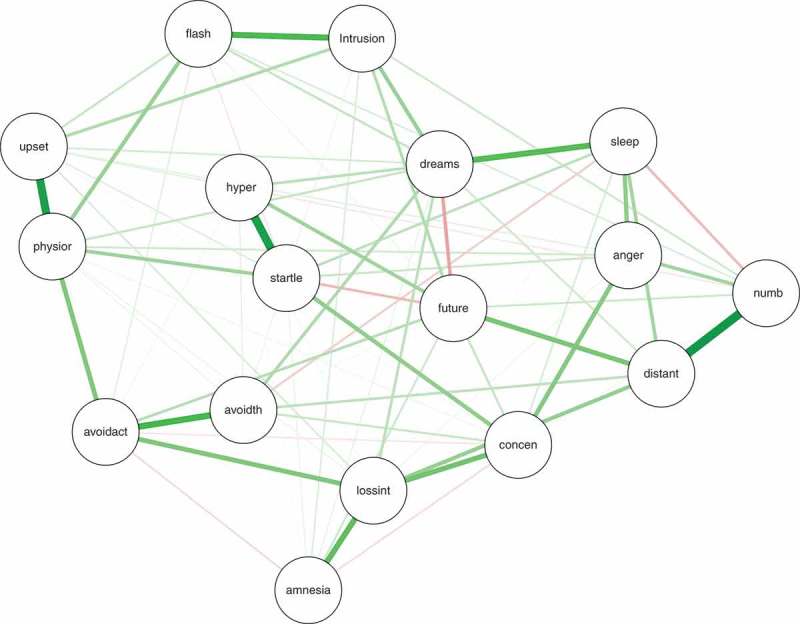
Regularized partial correlation network returned via the graphical LASSO depicting associations between pairs of PTSD symptoms. The 17 PTSD symptoms are: *intrusion* (intrusive memories, thoughts, or images of the trauma), *dreams* (traumatic dreams), *flash* (flashbacks), *upset* (at reminders of the trauma), *physior* (physiological reactivity in response to reminders of the trauma), *avoidth* (avoid thoughts and feelings about the trauma), *avoidact* (avoid activities reminiscent of the trauma), *amnesia* (difficulty remembering important aspects of the trauma), *lossint* (loss of interest in previously enjoyed activities, *distant* (feeling distant or cut off from others), *numb* (emotionally numb), *future* (future foreshortening), *sleep* (difficulty falling or staying asleep), *anger* (feeling irritable or having angry outbursts), *concen* (difficulty concentrating), *hyper* (hypervigilant), and *startle* (exaggerated startle).

The regularized partial correlation network returned by the graphical LASSO appears in Figure 1. Strong edges are apparent between feeling distant from others and emotional numbness; between exaggerated startle and hypervigilance; among anger, difficulty sleeping, and concentration problems; loss of interest in previously enjoyed activities and concentration problems; flashbacks and intrusive thoughts; and dreams about the trauma and disturbed sleep. There were several smaller negative edges (e.g. between dreams about the trauma and future foreshortening). We also bootstrapped the confidence regions of the edge weights, providing an estimate about precision of the edge weights in the network (see Figure S1 in the Supplemental data). Figure S1 shows that only the 12 thickest edges depicted in Figure 1 (e.g. startle–hypervigilance, emotional numbing–feeling distant from others; flashbacks–intrusive thoughts) are reliably different from zero.

**Figure 2.**
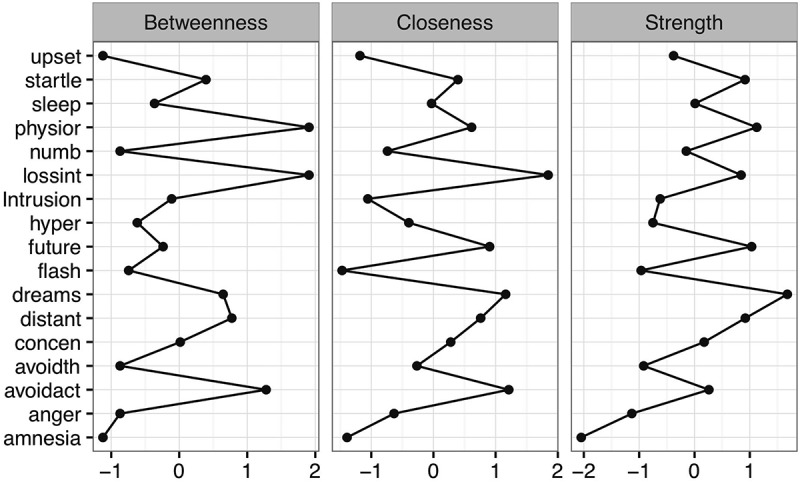
*z*-scored centrality metrics (betweenness, closeness, strength) for each PTSD symptom.

The *z*-scored centrality indices appear in Figure 2. Physiological reactivity to reminders of the trauma, dreams about the trauma, and loss of interest in previously enjoyed activities were often among the highest centrality symptoms across indices. Difficulty remembering aspects of the trauma exhibited consistently low centrality across indices. Taken together, these data suggest that dreams about the trauma, loss of interest in previously enjoyed activities, and physiological reactivity may be especially important to the maintenance of PTSD symptoms arising from CSA. However, our stability analyses suggest significant caution is warranted when interpreting these findings. The results of this analysis appear in the Supplemental data (Figure S2). In our person-dropping stability analysis, we found CS-coefficients of .13, .05, and .13 for our betweenness, closeness, and strength centrality metrics, respectively. Each of these values is below the recommended minimum threshold of .25, suggesting that our centrality estimates are unstable. Consistent with this finding, using the bootstrapped difference test in *bootnet*, we found that there were no significant differences between nodes for any of the three centrality indices (see Supplemental data, Figure S3).

Finally, the means, standard deviations, skewness values, and strength centrality metrics appear in Table S1 (see Supplemental data). Because the two-tailed Pearson correlation between the standard deviation and strength centrality was nonsignificant, *r*(15) = −0.11, *p *> .68 (and, indeed, negative), differential variability across symptoms does not pose a problem for interpreting a symptom’s strength centrality (see Terluin et al., 2016). The two-tailed Pearson correlation between skewness and strength centrality was likewise nonsignificant, *r*(15) = .12, *p* = .64.

### 2.2. DAG

**Figure 3.**
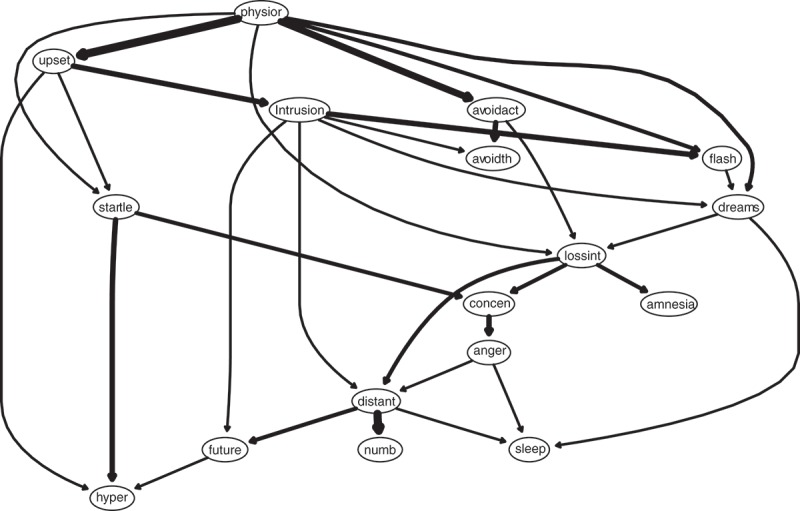
Directed acyclic graph (DAG). Edge thickness signifies the probability of prediction is in the direction depicted.

Figure 3 depicts the DAG arising from the averaging of the 10,000 bootstrapped networks whereby edge thickness signifies confidence that direction of prediction (and potentially causation) flows in the direction depicted in the graph. Several features are notable. First, physiological arousal in response to reminders of the trauma figures very prominently, consistent with Pavlovian fear conditioning theories of PTSD. It directly predicts being upset by reminders, flashbacks, traumatic dreams, and avoidance of activities reminiscent of the trauma. It also directly predicts exaggerated startle responses, and loss of interest in previously enjoyed activities. These findings suggest that extinguishing these physiological reactions to reminders may be the key to treating PTSD symptoms, at least among adults reporting sexual abuse as children. Feeling distant from other people, sleep difficulties, and numbness are downstream symptoms seemingly dependent on other symptoms in the network.

Figure S4 depicts the identical DAG except that edge thickness in this graph signifies how important an edge is to the model; the thicker an edge, the more vital it is to model fit (see Supplemental data).

### 2.3. Comparison of networks

To facilitate comparison between the two networks, we recalculated the regularized partial correlation network for participants for whom there were no missing data (i.e. the same participants whose data figured in the DAG analyses; *n *= 165). The DAG was notably sparser (31 non-zero edges) than the regularized partial correlation network (72 edges). Among the 31 edges present in the DAG, all were also present in the regularized partial correlation network. The latter included an additional 41 edges not present in the DAG. Notably, the 31 edges in the regularized partial correlation network that were also present in the DAG were each positive and had markedly greater mean edge weight (*M* = .20, *SD* = .12) than did the additional 41 edges that were absent from the DAG (*M* = .03, *SD* = .07) where there was a mix of positive and negative edges. In other words, the edges identified in the DAG tended to be strong edges in the regularized partial correlation network. Accordingly, these findings suggest that the DAG and regularized partial correlation network were in broad agreement about the most important edges in the network.

## 3. Discussion

Our study is the first concerning PTSD symptoms in adults reporting a history of CSA, and the latest in a series of network studies on PTSD beginning with one on earthquake survivors (McNally et al., 2015), We conducted network analyses by using two distinct approaches to characterizing the associations among symptoms: a regularized partial correlation approach and a Bayesian network approach.

### 3.1. Regularized partial correlation network

Regularized partial correlation networks have rapidly become a prime method for detecting possible causal relationships among elements of psychopathology systems. There are, of course, limitations to this approach, including the widely known risks in inferring causality from correlational data (e.g. Maurage, Heeren, & Pesenti, 2013). Two additional concerns have been recently raised. First, because edges reflect a type of correlation (regularized partial), inferences about correlation-based centrality metrics (e.g. strength centrality) may be distorted if nodes differ dramatically in terms of their variability in the study group (Terluin et al., 2016). In a study where most participants score very low (or high) on a symptom measure, such skewness can compress variability for a symptom, rendering it difficult to detect a correlation between it and other symptoms in the network. For example, the PTSD symptom referring to difficulty recalling important aspects of the trauma (‘amnesia’) was characterized by very low centrality metrics in our study as well as in other PTSD studies (Armour et al., 2017; Bryant et al., 2017; McNally et al., 2015; Mitchell et al., 2017). In our data, this symptom did exhibit a notably positive skew, potentially contributing to its low centrality. However, overall, we did not find evidence that restricted variability distorted estimates of our strength centrality metrics (see Table S1 in Supplemental data). Indeed, the positive skew for the amnesia symptom most likely reflects that fact trauma survivors encode and hence remember their trauma all too well (McNally, 2003, pp. 105–124), such that trauma survivors seldom endorse experiencing this symptom (Rubin, Berntsen, & Bohni, 2008). Hence, its low centrality is unlikely to be a statistical artefact arising from restricted variability.

Second, researchers have raised concerns about the accuracy, robustness, and replicability of edge weights and centrality metrics (e.g. Terluin et al., 2016). For many networks (e.g. Boston’s subway system), the edges that connect the nodes are directly observable (e.g. tracks between two train stations). In contrast, the edges in mental disorder networks are not directly observable and hence require estimation, thereby introducing the possibility of inaccurate or unstable estimates. Our stability and robustness analyses suggest that the findings from our regularized partial correlation network should be interpreted with caution. Our person-dropping analyses suggest that removing even a small portion of the sample (<15%) could lead to significant changes in which nodes are identified as being central to the network. As a consequence of this instability, our bootstrapped differences test suggests we cannot confidently conclude that any node in the network is significantly more central than any other. Similarly, our bootstrapping analyses produced wide confidence intervals around our edge weight estimates, suggesting that any conclusions from our network structure should be interpreted with caution. The low stability and wide confidence intervals around our edge estimates may be due, at least in part, to our small sample size (*N *= 179) relative to the number of parameters estimated in our analysis (136). Indeed, concerns about robustness of estimated edges and centrality metrics may be largely attributable to relatively small sample sizes in many studies. When many parameters require estimation, the number of participants one needs to estimate with confidence must be very large. However, it could also have arisen due to a high level of heterogeneity within the sample, such that small changes in the sample produce differences in the network. This possibility underscores the importance of intra-individual network analyses that estimate the structure of PTSD within individuals as an additional source of information about the structure of the PTSD network. In addition, these findings illustrate the importance of assessing the accuracy and stability of network parameter estimates.

### 3.2. Bayesian network

Perhaps the most salient feature of the DAG is its suggestion that symptoms of intrusive re-experiencing, especially physiological and emotional reactivity to reminders of the trauma, act as driving causal forces in the disorder. Conversely, symptoms of avoidance, loss of interest, concentration difficulty, anger/irritability, social disconnection, and emotional numbness, appeared to arise as direct or indirect consequences of those symptoms.

The appeal of Bayesian networks is their promise to disclose potentially causal links among nodes, even in cross-sectional data sets. Yet such promise comes with key assumptions that require satisfaction. To infer causation from such cross-sectional data, one must be confident that no important variables have been omitted from the network. Obviously, if a key variable driving symptoms is not part of the input, one cannot detect its causal influence. For example, the criteria set for DSM-5 PTSD has symptoms that were not present in the DSM-IV, including persistent negative emotions (e.g. shame, guilt, fear, anger, horror). Indeed, intrusive memories may prompt intense feelings of shame and guilt that may foster perceived social disconnection and trauma-related avoidance (Lee, Scragg, & Turner, 2001), especially among adult survivors of CSA (Clancy, 2009). In fact, two recent network studies on DSM-5 PTSD revealed that persistent negative emotional states had markedly high centrality (Armour et al., 2017; Mitchell et al., 2017). Yet this symptom was missing from our DSM-IV-based study. In future studies taking a Bayesian approach, it will be crucial to incorporate these negative emotional states into the analysis. Regrettably, though, DSM-5 groups five different emotions under this single symptom. These distinct emotions almost certainly differ in their cognitive and behaviour correlates, and likely play different roles in the PTSD network. Accordingly, they should be disaggregated and assessed separately.

Another potential limitation of our Bayesian analysis is that it disallows cycles whereby activation originating from a symptom activates other symptoms that then loop back to influence the original symptom. That is, there are no feedback loops in a DAG; activation flows only in one direction. For example, we found that becoming physiologically reactive to reminders of the trauma activates exaggerated startle. Yet it is possible that the relation between startle and physiological arousal may work both ways. As Figure 3 illustrates, the edge running from physiological arousal to startle is quite thin relative to other edges. A thin edge in a DAG, depicting the probability of edge direction, indicates that the direction reversed in a large minority of the bootstrapped networks. Accordingly, a thin edge in this type of DAG suggests that the direction of causation between two symptoms may go both ways (i.e. a possible ‘hidden’ cycle within an acyclic graph). Conversely, the thicker the edge in this type of DAG, the more confident we can be about inferring causal direction.

On the other hand, such sleuthing would not uncover cycles of length greater than two (e.g. *i *→ *j *→ *h *→ *i*) and there may be such cycles in the PTSD network (e.g. thoughts about the trauma activates intense physiological responses followed by efforts to avoid those thoughts, efforts that, in turn, have the ironic effect of increasing the frequency of thoughts about the trauma). Indeed, such cycles may play a critical role in the posited self-reinforcing nature of the PTSD network (McNally et al., 2015). In future studies, it will be important to evaluate how robust the analyses performed here are to violations of the assumption of acyclicity and it will be especially valuable to explore approaches for estimating directed cyclic graphs capable of detecting feedback loops among the symptoms of PTSD.

Finally, while double-checking our work, we discovered that our DAG analyses produced somewhat different results between operating systems (e.g., Windows 7 versus macOS Sierra) and between different versions of the same operating system (e.g., macOS El Capitan [version 10.11.6] versus macOS Sierra [version 10.12.5]). In contrast, re-running the analysis on different workstations using the same version of the same operating system produced equivalent DAGs. Neuroimaging researchers have likewise discovered that different operating systems can produce somewhat different results in their similarly computationally-intensive procedures (e.g. Gronenschild et al., 2012). As Gronenschild et al. (2012) observed, because it is unclear which operating system produces the more accurate results, investigators should avoid changing operating systems in the middle of a study and they should report details of their operating system and workstation when publishing their results. For the findings reported in this article, we used a MacBook Pro laptop computer [CPU = 2 x 2.9 GHz Intel Core i5; RAM = 16 Go 1867 MHz DDR] running the macOS Sierra [version 10.12.5] operating system.

## 4. Conclusion

PTSD is quickly becoming a major subject for network analytic approaches to psychopathology. A critical first step is to map the structure of relationships among PTSD symptoms, especially across different trauma types (e.g. CSA, combat, natural disaster). Confidence in our conclusions will grow to the extent that diverse network methods, each with its own strengths and limitations, converge empirically. To our knowledge, this study is the first to apply Bayesian methods to elucidate PTSD as a possible causal system. The networks produced by these analyses identify plausible symptom-symptom relations warranting further scrutiny. Accordingly, Bayesian analysis provides another method for the network analysis toolkit.

## Supplementary Material

Supplementary material

## Highlights

- We conducted network analyses of PTSD symptoms in adult survivors of childhood sexual abuse.
- We computed a graphical LASSO depicting regularized partial correlations between symptom pairs.
- Using a Bayesian approach, we computed a directed acyclic graph.
