# Supplementary material for: A Bayesian network analysis of posttraumatic stress disorder symptoms in adults reporting childhood sexual abuse
Source: Eur J Psychotraumatol. 2017 Jul 15;8(sup3):1341276. doi: 10.1080/20008198.2017.1341276 (PMC5632780; doi:10.1080/20008198.2017.1341276)
Supplement: Supplementary material [file ZEPT_A_1341276_SM7185.zip › McNally, Heeren, & Robinaugh EJP SUPPLEMENTARY revised JUNE 26 2017.docx]

**Supplementary Materials**

A Bayesian Network Analysis of Posttraumatic Stress Disorder Symptoms

in Adults Reporting Childhood Sexual Abuse

Richard J. McNally, Ph.D.

Harvard University

Alexandre Heeren, Ph.D.

Harvard University

and Université Catholique de Louvain

Donald J. Robinaugh, Ph.D.

Massachusetts General Hospital

and Harvard Medical School

**Network stability and robustness of the graphical LASSO**

To evaluate the robustness of our findings, we estimated both the accuracy of the edge weights and the stability of the centrality metrics for the graphical LASSO. To estimate the accuracy of the edge weights, we used a non-parametric bootstrap approach to calculate the 95% confidence intervals (CIs) for the edges by sampling the data 10,000 times (with replacement), thereby generating a distribution of edge weights. We accomplished this via the *R* package *bootnet* (Epskamp, Borsboom, & Fried, 2016).

To evaluate the stability of the centrality metrics, we implemented a subset (person-dropping) bootstrap procedure (Costenbader & Valente, 2003). We repeatedly correlated the centrality metrics from the full, original dataset with the centrality metrics calculated from subsamples comprising progressively fewer participants. If the correlations do not drop substantially during these procedures, then we can be reasonably confident that conclusions about the importance (centrality) of symptoms in the network are sound. Using the *R* package *bootnet* (Epskamp et al., 2016), we computed 10,000 bootstrap samples.

Figure S1 depicts the 95% confidence intervals (CIs) for the bootstrapped edge weights (i.e., regularized partial correlation coefficients between symptom pairs). The red line shows the values of the original edge weights from the largest to the smallest depicted from the top to the bottom of the figure. If the gray horizontal line does not cross the vertical line signifying a partial correlation of zero, then we can be confident that the edge does, indeed, differ from zero. Only about the dozen largest edges meet this criterion for stability.

Figure S2 illustrates the average correlation between the ordering of symptoms according to their centrality in the original network with the orderings after we randomly dropped a progressively higher proportion of the sample. We found CS-coefficients of .13, .05, and .13 for our betweenness, closeness, and strength indices, respectively, suggesting that dropping less than 15% of our participants leads to a non-trivial number of instances ( > 5% of samples) in which each of the centrality indices are no longer strongly correlated (*r* ≥ .7) with the full sample.

Figure S3 presents the results from our bootstrapped differences tests for the three centrality indices. Gray boxes indicate nodes did not significantly differ from one another. We found that there were no significant differences between nodes for any of the three indices.

Table 1 shows the means, standard deviations, skewness, and strength centrality values for the 17 PTSD symptoms. The two-tailed Pearson correlation between the standard deviation and strength centrality was nonsignificant, *r*(15) = -0.11, *p* = .68). Had a significant positive correlation emerged, this would suggest that a symptom’s importance in the network was affected by its variability. The skewness metric indicated that symptoms exhibited some degree of positive skew, but most of these values were modest, dreams and flashbacks concerning the trauma, excepted. Taken together, these data suggest the differential variability across symptoms does not complicate interpretation of a symptom’s strength centrality.

**Complementary analyses related to the DAG**

Figure S4 depicts the DAG whose edge thickness signifies how important an edge is to the model; the thicker and edge, the more vital it is to model fit.

***Figure S1.*** Bootstrapped edge weights (regularized partial correlations between symptom pairs). The red line indicates the sample values and the gray area the 95% confidence intervals.

***Figure S2.*** Average correlation between centrality metrics in the bootstrapped samples and the original centrality metrics as a function of the percentage of participants in the bootstrapped sample.


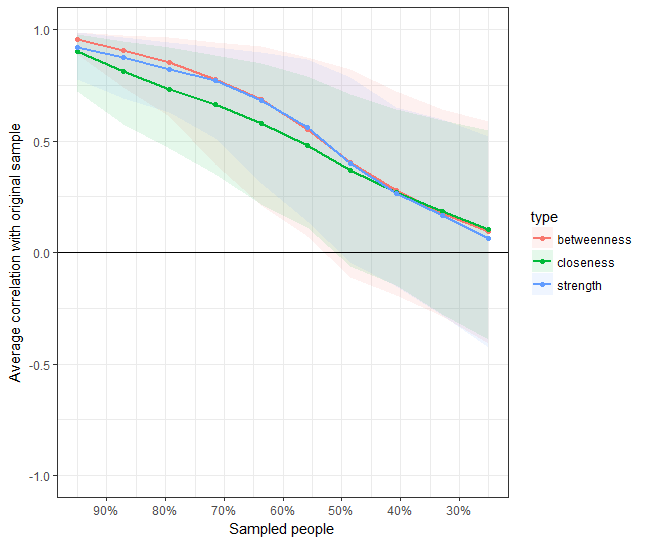


***Figure S3***. Bootstrapped differences test for centrality indices.


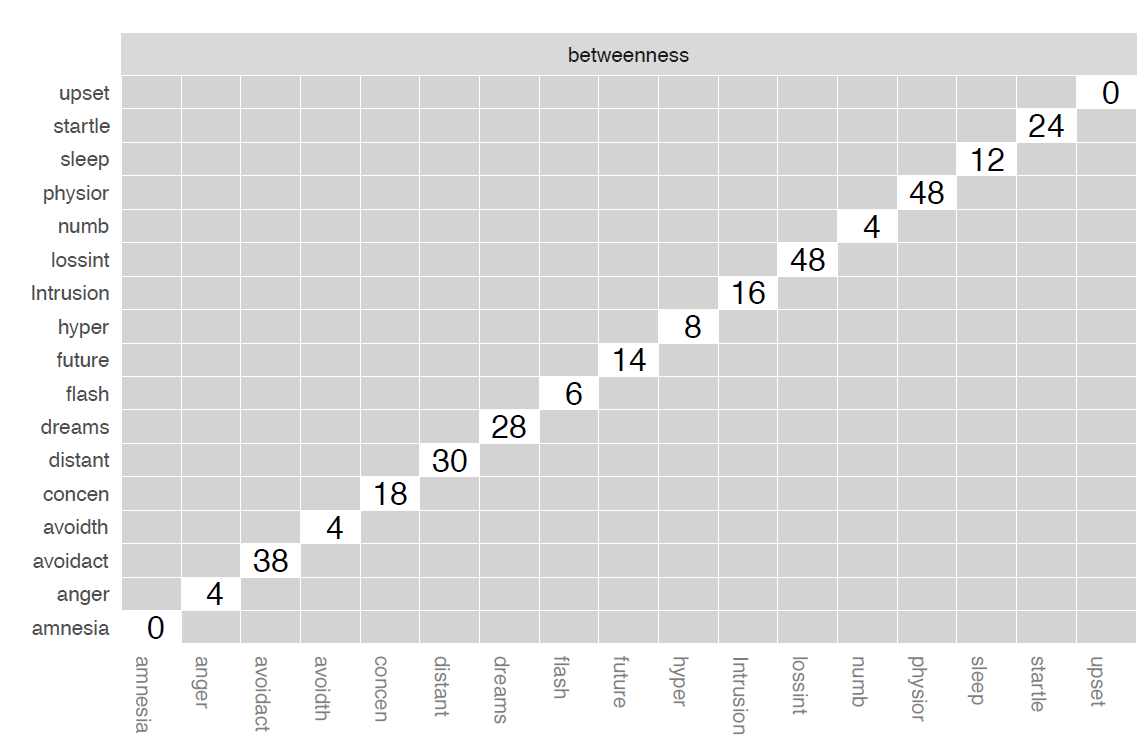


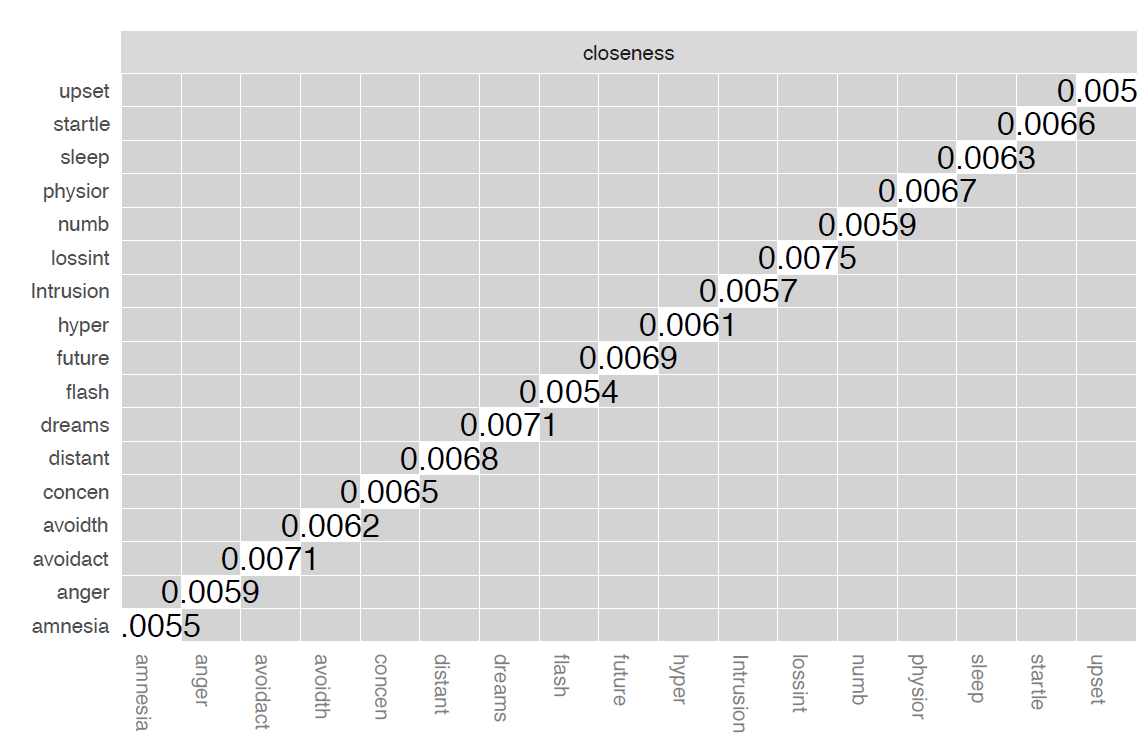


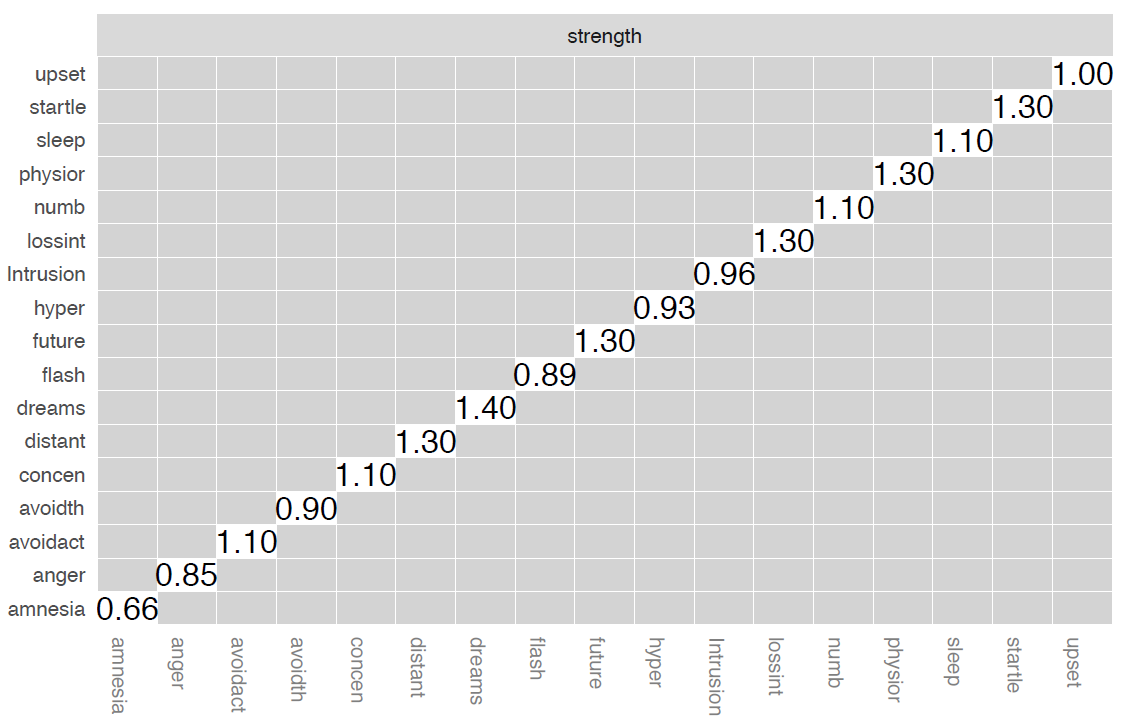


***Table S1.*** Means, standard deviations, skewness, and strength centrality for each symptom.

Symptom M SD Skewness Centrality

Intrusions 1.08 1.14 0.55 0.96

Dreams 0.63 0.98 1.35 1.45

Flashbacks 0.72 1.00 1.15 0.89

Upset at Reminders 1.16 1.15 0.38 1.01

Physio Reactive 0.86 1.14 0.89 1.33

Avoid thoughts 1.23 1.30 0.35 0.90

Avoid activities 0.95 1.20 0.72 1.15

Amnesia 0.88 1.18 0.89 0.66

Loss of interest 0.88 1.14 0.90 1.27

Distant 1.42 1.27 0.08 1.29

Numb 1.44 1.29 0.06 1.06

Future foreshortening 1.16 1.28 0.45 1.31

Sleep 1.45 1.32 0.05 1.09

Anger 1.08 1.19 0.55 0.85

Concentration 1.18 1.19 0.37 1.13

Hypervigilance 1.25 1.26 0.32 0.93

Startle 1.10 1.23 0.54 1.28

-------------------------------------------------------------------------------------------------------------

*Note.* See Figure 1 caption for explanation of symptom abbreviations.

This table shows the means, standard deviations, skewness, and strength centrality values for the 17 PTSD symptoms. The two-tailed Pearson correlation between the standard deviation and strength centrality was nonsignificant, *r*(15) = -0.11, *p* = .68). Had a significant positive correlation emerged, this would suggest that a symptom’s importance in the network may be affected by its variance. The skewness metric indicated that symptoms exhibited some degree of positive skew, but most of these values were modest, dreams and flashbacks concerning the trauma, excepted. Taken together, these data suggest the differential variability across symptoms does not complicate inter pretation of a symptom’s strength centrality.

***Figure S4***. Directed acyclic graph (DAG). Edges signify the importance (BIC value) of the edge to model fit.

**References**

Costenbader, E., & Valente, T. W. (2003). The stability of centrality measures when

networks are sampled. *Social Networks, 25*, 283-307.

Epskamp, S., Borsboom, D., & Fried, E. I. (2016). Estimating psychological networks and their stability: A tutorial paper. *Arxiv Preprint* (*ID1604.08462*), 1-34.
